# From waste to wealth: effects of nine agroforestry wastes on Stropharia’s traits & yield

**DOI:** 10.1515/biol-2025-1189

**Published:** 2026-01-26

**Authors:** Meng Shen, Guoying Lv, Weiqiang He, Ning Wang, Ruisen Wang, Xinhua Quan, Ye Yuan, Xiangtan Yao

**Affiliations:** Jiaxing Academy of Agricultural Sciences, 314001, Jiaxing, China; Zhejiang Academy of Agricultural Sciences, 310021, Hangzhou, China; Jiaxing Vocational & Technical College, 314001, Jiaxing, China; Hebei Province Shijiazhuang Agricultural Technology Promotion Center, 050050, Hebei, China

**Keywords:** agricultural straw, culture formula, economic effectiveness, fruit tree branch, resource utilization

## Abstract

To broaden *Stropharia rugosoannulata* cultivation substrates, nine agricultural/forestry wastes were tested, evaluating fruiting body count, weight, and total yield, with factors influencing yield also investigated. Fig branches performed best: 16.5 % grade A fruiting bodies, 5.11 kg/m^2^ total yield, and 232,000 yuan/hm^2^ net profit. Grape stumps, pear and peach branches were also suitable, with yields 5.38, 4.64, 4.62 kg/m^2^ respectively. Waste sawdust had 16.2 % grade A but limited economic benefits. Willow branches (9.5 % grade A), crape myrtle branches (8.4 %), soybean stalks (12.5 %), and corn stalks (4.04 kg/m^2^ total yield) were suboptimal, needing reduced use. Correlation analysis showed first harvest reflects suitability; high lignocellulose materials enhance individual mushroom quality.

## 1 Introduction

*Stropharia rugosoannulata*, commonly referred to as the wine cap mushroom, is an edible fungus classified under the order Agaricales, family Strophariaceae, and genus *Stropharia* [1]. It is celebrated for its fragrant aroma and crisp texture [2]. This species possesses a complete cellulase system and secretes laccases [3], enzymes capable of degrading lignin, endowing it with a strong capacity to break down lignocellulose. This capability enables it to adapt to various cultivation materials, including non-grain straw, broadleaf hardwood sawdust, orchard pruning, and crop straw [4], [5], [6]. Notably, it can be cultivated using unprocessed materials [7]. Additionally, the cultivation methods *for S*. *rugosoannulata* are diverse, encompassing open-field, facility-based, and under forest cultivation [8], [9], [10], each with distinct advantages [11]. Numerous studies have explored under forest cultivation using orchard prunings, a practice that facilitates the recycling of branches back into the soil while enabling intercropping and crop rotation [10]. China’s circular economy development plan underscores the significance of advancing circular economic practices to achieve efficient and sustainable resource utilization. Agricultural and forestry waste, particularly in Zhejiang Province, constitutes a valuable biological resource. In 2018, the province generated approximately 10 million tons of crop straw, with rice straw being the primary component. Comprising lignin, cellulose, and hemicellulose [12], these residues necessitate diverse reutilization strategies due to the recalcitrant nature of lignin and cellulose. Edible fungi, such as *S*. *rugosoannulata*, play an indispensable role in the circular utilization of these wastes by effectively decomposing these complex substances [13], [14], [15]. This not only enhances the utilization efficiency and added value of post-harvest straw but also aligns with the broader objectives of circular agriculture and ecological sustainability, which are imperative for the future of agriculture [16]. The cultivation of *S*. *rugosoannulata* is particularly advantageous due to its diverse cultivation methods and high success rate, making it an optimal choice for reusing various agricultural and forestry wastes. By integrating the cultivation industry of this mushroom as a central component, extending the agricultural and forestry industrial chains, adjusting planting structures, and achieving efficient recycling of agricultural and forestry waste is possible [17].

In 2023, Zhejiang Province had 291,700 ha of fruit trees cultivated, 6,089,100 ha of forestland, 81,000 ha of soybean, and 28,000 ha of corn planted. Specifically, pear trees cover 16,190 ha, and peach trees cover 28,950 ha. Jiaxing City focuses on the grape, peach, and pear industries. Most fruit trees require pruning during cultivation, as do commercial forests and roadside trees in their management, resulting in a large quantity of waste branches annually. In contrast to crop straw, fruit tree branches cannot be directly returned to the field and are often burned [18] or discarded. Branches from fruit trees can be collected easily and are rich in lignocellulose, making them excellent raw materials for cultivating edible fungi [16], 19].

In this study, we investigated the feasibility of utilizing nine common agricultural and forestry waste materials from the Jiaxing region as novel supplements for substrate mixtures in cultivating *S*. *rugosoannulata*. Initially, the nutritional components of waste sawdust from *Pleurotus ostreatus* and branches from pear, grape, fig, peach, crape myrtle, and willow trees, along with soybean and corn stalks, were analyzed. Extracts from these materials were subsequently used to culture the mycelia of *S*. *rugosoannulata*. The cultivation process involved using rice straw and rice husk as the primary substrates, supplemented with nine types of agricultural and forestry waste, in a controlled greenhouse environment. The growth data of *S*. *rugosoannulata* were meticulously recorded, including metrics such as the average weight per mushroom, the number of fruiting bodies, and the overall yield. These indicators were used to perform a correlation analysis and calculate comprehensive economic benefits, identifying the most suitable agricultural and forestry waste materials for local cultivation in Jiaxing. The findings provided new methods for utilizing agricultural and forestry waste and selecting substrates for cultivating *S*. *rugosoannulata*.

## 2 Materials and methods

### 2.1 Test material

The strain of *S*. *rugosoannulata* used in this experiment was provided by the Zhejiang Academy of Agricultural Sciences and was designated as ZJR 19 (with patent deposit number CCTCC NO: M20232517), and it was preserved in liquid nitrogen. The formula of the strain substrate was as follows: 87 % wood chips, 6 % wheat bran, 6 % soybean meal, and 1 % lime, with a moisture content of 63 %. Each polypropylene plastic bag was filled with 200 g of dry culture material, sterilized at 121 °C for 2 h, cooled, inoculated with activated mycelia, and then cultured at 23 °C until fully colonized.

**Figure 1:**
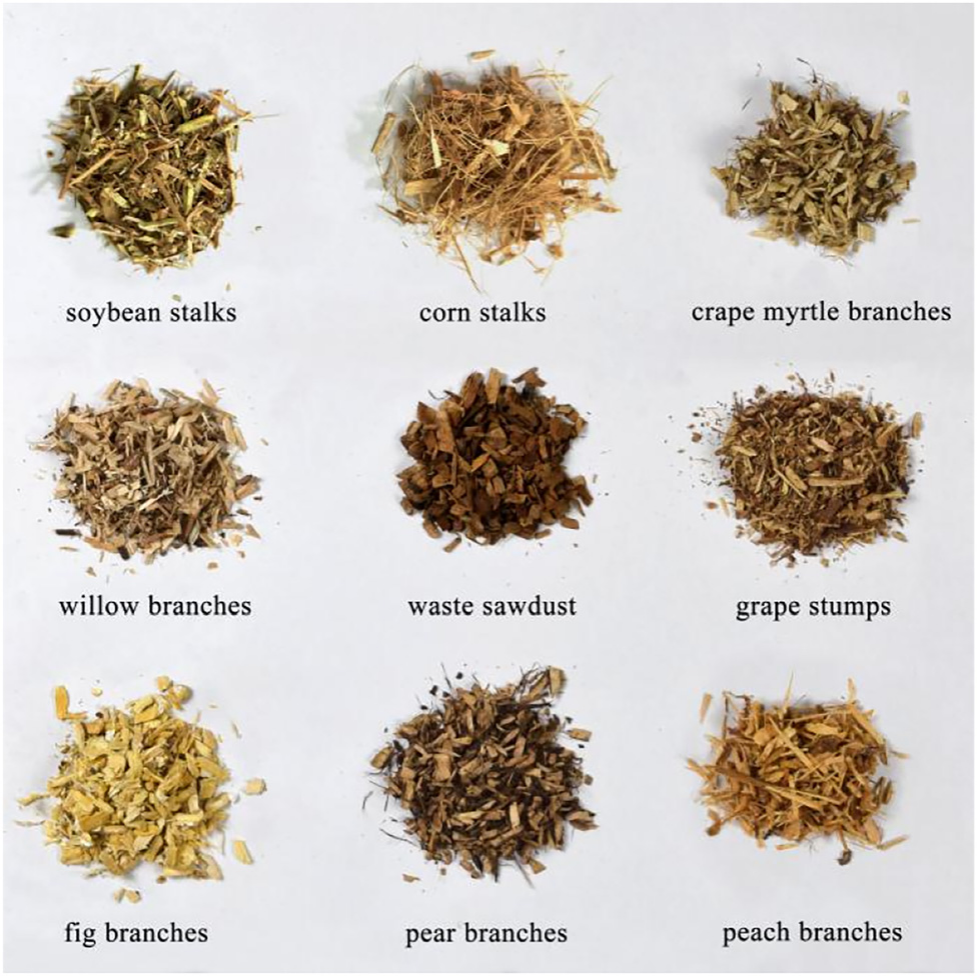
The nine types of crushed agricultural and forestry waste materials.

Nine types of agricultural and forestry waste were used (Figure 1): soybean stalks, corn stalks crape myrtle branches (*Lagerstroemia indica* L.), willow branches (*Salix babylonica* L.), waste sawdust, grape stumps (*Vitis vinifera* L.), fig branches (*Ficus carica* L.), pear branches *(Pyrus* spp.), and peach branches (*Prunus persica* (L.) Batsch). The waste sawdust consisted of contaminated substrate from *P*. *ostreatus* cultivation, which was crushed and fermented. The pear, fig, peach, crape myrtle, and willow branches were pruned during the current year, while the grape stumps were discarded during variety replacement. All agroforestry wastes were air-dried and crushed into wood chips with a diameter of 4–8 mm.

### 2.2 Experimental location and time

The experiment was conducted at the Jiaxing Academy of Agricultural Sciences, which is located in the East Asian monsoon region; the average annual temperature is 15.9 °C, and the average annual precipitation is approximately 1,193.2 mm. The study site featured acidic paddy soil, and cultivation was carried out in a greenhouse (25 m × 8 m × 3 m). The experiment ran from October 2, 2023, to May 15, 2024.

### 2.3 Cultivation methods

The cultivation substrate consisted of 40 % agricultural and forestry waste, 30 % rice straw, and 30 % rice husk. Before sowing, the greenhouse was aerated for one week, and the soil was tilled 2–3 times via a rotary tiller to create 27 plots, each measuring 3.2 m in length and 0.6 m in width. The materials were pre-moistened, thoroughly mixed, and then spread into the plots. The substrate was applied at a rate of 7.5 kg/m^2^ of dry material, and the spawn was used at 600 g/m^2^. After sowing, cover the soil and sprinkle crushed straw to keep the surface moist. Ventilate for 8 h daily and adjust the substrate humidity to 70 % before mushroom emergence. Use a shade net over the greenhouse and maintain soil moisture during mushroom growth, adjusting the substrate humidity back to 70 % when water conditions change. A randomized block design was used, with each treatment replicated three times.

### 2.4 Preparation of the sawdust boiled solution and determination of the mycelial growth rate

Cultivation of mycelial blocks of uniform age: Mycelia from the test tube were transferred to a Petri dish for activation and incubated at 25 °C until they covered the entire dish. Uniformly sized mycelial blocks were then removed from the colony edge for the experiment.

To prepare wood chip extract: 20.0 g of wood chips were boiled in 1 L of water for 20 min and then filtered through gauze. The filtrate was concentrated to a final volume of 100 mL for subsequent use. PDA media were then prepared with varying concentrations of wood chip extract: 0 mL (control), 5 mL, 20 mL, and 35 mL. In each case, the wood chip extract was added to PDA to achieve a total final volume of 125 mL. Using a pipette, 12 mL of each medium was transferred to Petri dishes. After cooling, mycelial plugs were inoculated at the center of each dish. Colony diameters were measured on the third and fifth days postinoculation, with each treatment replicated five times.

### 2.5 Index record

First harvest: The period from inoculation to the first harvest. Total harvest days: The total number of days that mushrooms are available for harvesting in the field. Harvest period: The duration from the first to the last harvest. Grade A mushrooms: Fruiting bodies with a stem diameter of ≥35 mm. Grade B mushrooms: Fruiting bodies with a stem diameter of <35 mm.

### 2.6 Methods for sample preparation and index determination

Water content: The water content of the samples was determined based on the national standard “Method for Determining the Water Content of Wood GB/T 1931–2009.”

Water-holding capacity [20]: This refers to the maximum amount of water that the cultivation material can retain. A 2.000 g sample was placed in a 50 mL beaker with 45 mL of distilled water and soaked for 24 h at 25  ± 2 °C. The mixture was then centrifuged at 3,600 r/min for 20 min, k held in an inverted position for 2 h to drain excess liquid, and weighed.

Total carbon and total nitrogen contents: Samples were dried to a constant weight at 45 °C, crushed, sieved through a 100-mesh sieve, and then stored in a dry environment. The total nitrogen content was determined employing the standard Kjeldahl method, and the protein content was estimated via the macro-Kjeldahl method with an SKD-1800 automatic Kjeldahl nitrogen analyzer, with a conversion factor of 6.25. The total carbon content was measured using a SerCon Integra two carbon-nitrogen isotope mass spectrometer. The contents of cellulose, hemicellulose, and lignin were determined following the procedures described in Xiong S’s study [21].

Dry matter content of fruiting bodies: Fresh fruiting bodies from the first flush were selected, and the mud-contaminated parts of the stipes were removed. The fresh weight of each sample was measured, followed by drying in an oven at 45 °C until a constant weight was reached. The dry weight was recorded, and the dry matter content was calculated.

### 2.7 Computational formula

The formula for calculating the water-holding capacity (V) is as follows:

(1) $$V=\frac{m_{w}-m_{d}}{m_{d}}$$

In the formula, m_w_ represents the wet weight of the culture material (g), and m_d_ denotes the dry weight of the culture material (g).

The formulae for calculating the profit of grade A mushrooms (PA, 10,000 yuan/hm^2^) and grade B mushrooms (PB, 10,000 yuan/hm^2^) are as follows:

(2) $$P_{A}=\frac{m_{a}\times p_{a}\times 0.6\times 667\times 15}{1.1\times 10000}$$

(3) $$P_{B}=\frac{m_{b}\times p_{b}\times 0.6\times 667\times 15}{1.1\times 10000}$$

Here, m_a_ represents the total yield of grade A mushrooms (kg/m^2^); p_a_ denotes the price of grade A mushrooms (30 yuan/kg); m_b_ indicates the total yield of Grade B mushrooms (kg/m^2^); and p_b_ signifies the price of grade B mushrooms (10 yuan/kg).

The formula for calculating the labor cost of mushroom picking (C, 10,000 yuan/hm^2^) is as follows:

(4) $$C=\frac{D\times w\times 7}{10000}$$

In the formula, D represents the number of days for harvesting mushrooms (in days), and w denotes the wage for mushroom harvesters (120 yuan per day, with an average of seven workers per hectare).

The formula for calculating net profit (Np, 10,000 yuan/hm^2^) is as follows:

(5) $$Np=P_{A}+P_{B}-C-S-M-Z$$

Here, P_A_ represents the profit from grade A mushrooms (10,000 yuan per hectare); P_B_ denotes the profit from grade B mushrooms (10,000 yuan per hectare); C represents the labor cost of mushroom harvesting (10,000 yuan per hectare); s represents the cost of mushroom spawn (3,100 yuan per hectare); M represents the cost of cultivation materials (1,600 yuan per hectare); and Z represents the cost of material preparation and loading (1800 yuan per hectare).

### 2.8 Statistical analysis

All experiments were conducted in triplicate, and the results were expressed as the mean ± standard deviation. Data analysis was performed using WPS Office for basic statistics, while IBM SPSS Statistics 23 was used for significance analysis and Pearson correlation analysis. Graphics were generated using OriginLab (Origin 2021). According to Duncan’s multiple range test, mean values within the same column for each oyster mushroom that were followed by the same letters were not significantly different at *p* < 0.05.

## 3 Results

### 3.1 Water content and water-holding capacity of different materials

After air-drying under natural conditions and storage, the water content of the nine materials, from highest to lowest, was as follows: willow branches > fig branches > corn stalks = peach branches > grape stumps > pear branches > waste sawdust = soybean stalks > crape myrtle branches(Table 1). All materials had a water content of less than 10 % after drying and storage. The water-holding capacity of the nine materials varied significantly (*p* = 0.00). It could be ranked from highest to lowest as follows: grape stumps = corn stalks > fig branches > pear branches = willow branches = waste sawdust > crape myrtle branches > peach branches > soybean stalks. The strongest water retention was observed in grape stumps, whereas the weakest water retention was observed in soybean stalks. This indicated that for the same weight of material used in substrate preparation, grapevines can incorporate more water. Materials with a relatively high WHC were easier to manage during mycelial growth. Additionally, in the later management stages, the grape stumps formula resulted in better water absorption, ensuring the necessary moisture for the growth of the *Stropharia* mushroom. When materials with poor water-holding capacity are used, attention must be given to the amount added and the proper proportion of materials with higher WHC.

**Table 1:** Water content and water-holding capacity of nine different materials.

Material	Water content (%)	Water-holding capacity
Waste sawdust	8.49 ± 0.12 f	4.17 ± 0.08 c
Pear branches	8.83 ± 0.02 e	4.24 ± 0.01 c
Grape stumps	8.97 ± 0.02 d	4.80 ± 0.02 a
Fig Branches	9.40 ± 0.05 b	4.56 ± 0.08 b
Peach branches	9.09 ± 0.02 c	3.76 ± 0.01 e
Crape myrtle branches	8.15 ± 0.05 g	3.95 ± 0.09 d
Willow branches	9.52 ± 0.06 a	4.20 ± 0.02 c
Soybean stalks	8.45 ± 0.07 f	2.85 ± 0.17 f
Corn stalks	9.17 ± 0.09 c	4.75 ± 0.05 a

The water-holding capacity is calculated using formula (1).

### 3.2 Carbon and nitrogen contents of different materials

In this experiment, all the fruit tree branches were twigs, with a relatively low total carbon content and a relatively high total nitrogen content. Waste sawdust shavings have a carbon content of 44.7 %, whereas willow branch shavings contain 43.55 % carbon, both of which are significantly greater than those of other wood shavings and straw (Table 2). The carbon content ranking was as follows: waste sawdust ≥ willow branches = rice straw ≥ peach branches ≥ crape myrtle branches = corn stalks = soybean stalks ≥ pear branches ≥ fig branches = grape stumps > rice husk. Agro-industrial waste contains various sugars and serves as a primary source of carbon [12]. The nitrogen content also differed among different wood shavings and straws, with soybean stalks having the highest nitrogen content and waste sawdust the lowest. The nitrogen content ranking was as follows: soybean stalks > pear branches = corn stalks > fig branches > peach branches > willow branches = crape myrtle branches = grape stumps = rice husk > rice straw = waste sawdust (Table 2). Waste wood shavings had the highest total carbon content (44.7 %) among the nine agricultural and forestry wastes but the lowest total nitrogen content (0.7 %), resulting in the highest carbon-nitrogen ratio(C/*N*) of 64:1. The C/*N* were as follows: waste sawdust > rice straw > crape myrtle branches > grape stumps > rice husk > willow branches > peach branches > fig branches > corn stalks > pear branches (Table 2).

**Table 2:** Carbon and nitrogen contents of the nine different materials.

Material	Total carbon content (%)	Total nitrogen content (%)	C/*N*
Waste sawdust	44.70 ± 0.92 a	0.70 ± 0.02 f	64∶1
Pear branches	39.58 ± 1.86 cde	1.33 ± 0.05 b	30∶1
Grape stumps	39.16 ± 0.97 de	0.96 ± 0.00 e	41∶1
Fig Branches	39.26 ± 1.96 de	1.22 ± 0.01 c	32∶1
Peach branches	41.88 ± 1.84 bc	1.16 ± 0.04 d	36∶1
Crape myrtle branches	41.43 ± 1.09 bcd	0.96 ± 0.02 e	43∶1
Willow branches	43.55 ± 1.52 ab	1.11 ± 0.03 e	39∶1
Soybean stalks	41.14 ± 0.07 bcd	2.59 ± 0.05 a	16∶1
Corn stalks	41.19 ± 0.07 bcd	1.35 ± 0.05 b	31∶1
Rice husk	38.10 ± 0.14 e	0.95 ± 0.05 e	40∶1
Rice straw	43.20 ± 1.40 ab	0.72 ± 0.04 f	60∶1

### 3.3 Hemicellulose, cellulose, and lignin contents of different materials

In the analysis of the nine materials, the hemicellulose content was ranked as follows: peach branches = rice straw = fig branches ≥ rice husk = corn stalks ≥ pear branches = soybean stalks ≥ crape myrtle branches = willow branches = waste sawdust = grape stumps (Table 3). The hemicellulose content in peach branches was 60.8 % greater than that in grape stumps. The cellulose content was ranked as follows: rice straw > corn stalks > rice husk > soybean stalks > crape myrtle branches = waste sawdust > grape stumps = pear branches > willow branches = fig branches > peach branches. The cellulose content in straw was significantly greater than that in branches, with straw having 149.6 % more cellulose than peach branches (Table 3). The lignin content decreased in the following order: willow branches = peach branches > pear branches = waste sawdust > grape stumps > rice husks = crape myrtle branches > corn stalks ≥ fig branches ≥ soybean stalks = rice straw. The lignin content reflects the degree of lignification in agricultural and forestry waste, with willow branches, peach branches, pear branches, and waste sawdust resulting in greater lignification. Specifically, the lignin content in the willow branches was 108.9 % greater than that in the soybean stalks. Among tree branches, fig branches had the lowest lignin content (17.17 %), which was similar to that of straw materials. When the total contents of the three components were considered, the ranking followed the order: rice husks > rice straw > corn stalks > peach branches > willow branches > pear branches > waste sawdust > crape myrtle branches > soybean stalks > grape stumps > fig branches. The combined proportion of hemicellulose, cellulose, and lignin in peach branches, corn stalks, rice straw, and rice husks was relatively high, exceeding 70 %. In contrast, fig branches and grape stumps had a lower combined proportion of these components, with each component having a value below 60 % (Table 3). Hemicellulose, cellulose, and lignin are the primary components present in most agricultural and forestry waste [12].

**Table 3:** Hemicellulose, cellulose, and lignin contents of nine different materials.

Material	Hemicellulose (%)	Cellulose (%)	Lignin (%)	All (%)
Waste sawdust	17.76 ± 2.32 c	16.55 ± 0.24 e	30.08 ± 0.43 b	64.40
Pear branches	20.85 ± 3.46 bc	15.05 ± 0.24 f	30.85 ± 0.99 b	66.76
Grape stumps	17.52 ± 2.19 c	15.08 ± 0.35 f	27.35 ± 0.64 c	59.96
Fig Branches	25.1 ± 2.24 a	14.03 ± 0.05 g	17.17 ± 0.55 ef	56.30
Peach branches	28.17 ± 0.86 a	12.63 ± 0.26 h	33.22 ± 0.81 a	74.02
Crape myrtle branches	19.61 ± 3.8 c	17.00 ± 0.02 e	24.96 ± 0.67 d	61.57
Willow branches	19.55 ± 2.19 c	14.41 ± 0.34 g	34.05 ± 0.52 a	68.01
Soybean stalks	20.58 ± 2.18 bc	24.30 ± 0.17 d	16.30 ± 0.64 f	61.19
Corn stalks	24.19 ± 0.99 ab	29.64 ± 0.14 b	17.56 ± 0.82 e	71.40
Rice husk	24.67 ± 1.22 ab	27.67 ± 0.41 c	25.62 ± 0.48 d	77.96
Rice straw	26.31 ± 1.83 a	31.53 ± 0.34 a	16.73 ± 0.67 ef	74.58

### 3.4 Effects of varying concentrations of nine different materials on the mycelial growth of *S*. *rugosoannulata*

**Figure 2:**
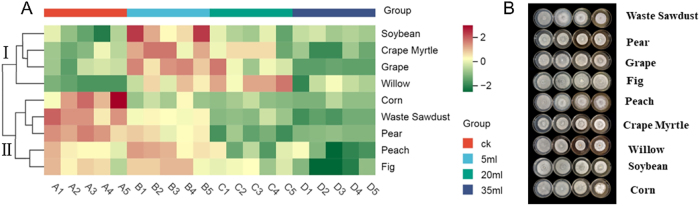
Effects of the decoction on the growth of *S*. *rugosoannulata* mycelium. (A) Heatmap of the growth rate of *S*. *rugosoannulata* mycelium. (B) Photographs depicting the density and whiteness of the mycelium.

The extracts from sawdust and straw can reduce the growth rate of *S*. *rugosoannulata* mycelia while increasing their density and whiteness. As illustrated in Figure 2, cluster analysis categorized the extracts from nine raw materials into two main groups. The first cluster (Ⅰ) included extracts from soybean stalks, crape myrtle branches, fig branches, and waste sawdust. When small quantities (5–20 mL) of these extracts were added, they initially accelerated the growth of *S*. *rugosoannulata* myceli*a*. However, as the quantity of extract increased, the growth rate decreased. Adding 5 mL of extracts from soybean stalks, crape myrtle branches, and fig branches significantly accelerated mycelial growth, whereas the effect was less pronounced at 20 mL. In contrast, adding 5–20 mL of miscellaneous waste sawdust extract consistently resulted in a significant increase in the growth rate. The second cluster (Ⅱ) comprised extracts from corn stalks, pear branches, peach branches, willow branches, and grape stumps. As the quantity of these extracts increased, the mycelial growth rate progressively decreased. The extract of corn stalks exhibited the most pronounced inhibitory effect, as the growth rate started slowing even with the addition of just 5 mL. Additionally, as the concentrations of extracts from soybean stalks, crape myrtle branches, fig branches, pear branches, peach branches, and grape stumps increased, the density and whiteness of *S*. *rugosoannulata* mycelium improved considerably. The decoctions of various wood sawdust types inhibited the mycelial growth of *S*. *rugosoannulata* [4]. While the decoction inhibited mycelial extension, it simultaneously induced denser mycelial growth, thereby reducing the overall rate of expansion. Additionally, some polyhydroxyphenols with good water solubility, as well as quinones generated after oxidation, are present in the decoction, which may affect the growth of mycelia [22], 23]. These inhibitory substances are present in relatively high concentrations in Cluster II.

### 3.5 Effects of agricultural and forestry waste on the production time of *S*. *rugosoannulata*

The C/*N* of the cultivation substrate changed when rice straw and rice husk were mixed (Table 4). Specifically, the substrate containing fig branches sawdust presented a C/*N* ratio of 40:1 and yielded the shortest first harvest of 42 days. The treatment with grape stumps material resulted in a C/*N* ratio of 45:1 and a first harvest of 46.5 days. Substrates containing pear branches, peach branches, willow branches, soybean stalks, and corn stalks presented C/*N* ratios ranging from 26:1 to 44:1, with the first harvest not significantly (*p* = 0.07) different from those of the grape stumps treatment. Treatments using waste sawdust and crape myrtle branches, with relatively high C/*N* ratios of 54:1 and 46:1, respectively, resulted in the longest first harvest times of 51.7 and 51.0 days. The total harvest days in the waste sawdust, soybean stalks, and corn stalks treatments extended beyond 90 days. In contrast, those in the peach branches and crape myrtle branches treatments were shorter than 70 days (Table 4). The peach branches treatment resulted in the shortest fruiting period of 63 days. The harvest period remained relatively consistent across all nine agricultural and forestry waste materials, averaging about 204 days.

**Table 4:** Production time of *S*. *rugosoannulata* cultivated with various agricultural and forestry wastes.

Material	Culture substrate C/*N*	First harvest (day)	Total harvest days (d)	Harvest period (d)
Waste sawdust	54∶1	51.7 ± 1.5 a	93.0 ± 6.2 a	205.0 ± 1a
Pear branches	39∶1	49.5 ± 3.5 ab	77.5 ± 13.5 bcd	204.5 ± 0.7 ab
Grape stumps	45∶1	46.5 ± 0.7 b	87.0 ± 0.0 abc	204.5 ± 2.1 ab
Fig Branches	40∶1	42.0 ± 1.4 c	87.5 ± 7.8 abc	204.5 ± 0.7 ab
Peach branches	42∶1	48.0 ± 1.4 ab	63.0 ± 1.4 e	204.5 ± 0.7 ab
Crape myrtle branches	46∶1	51.0 ± 1.7 a	67.7 ± 1.15 de	204.7 ± 0.58 ab
Willow branches	44∶1	48.0 ± 1.0 ab	74.3 ± 4.5 cde	205.0 ± 0.0 a
Soybean stalks	26∶1	47.7 ± 2.9 ab	90.3 ± 5.5 ab	204.3 ± 0.58 ab
Corn stalks	39∶1	50.5 ± 2.1 ab	91 ± 2.8 ab	203.0 ± 0.0 b

The C/*N* ratio of the substrate affects the first harvest time of edible fungi: a higher C/*N* ratio prolongs the first harvest [24]. However, the previous studies on the C/*N* ratio establish C/*N* gradients by adjusting the proportion of components without changing the substrate materials. In this study, the duration of the first harvest and the total number of harvest days may be intrinsically linked to the substrate composition and texture. A shorter first harvest might indicate a preference for *S*. *rugosoannulata*’s specific substrate characteristics, whereas a reduced number of total harvest days suggests a more concentrated fruiting pattern.

### 3.6 Effects of agricultural and forestry wastes on the pre-fruiting temperature of *S*. *rugosoannulata* culture materials

**Figure 3:**
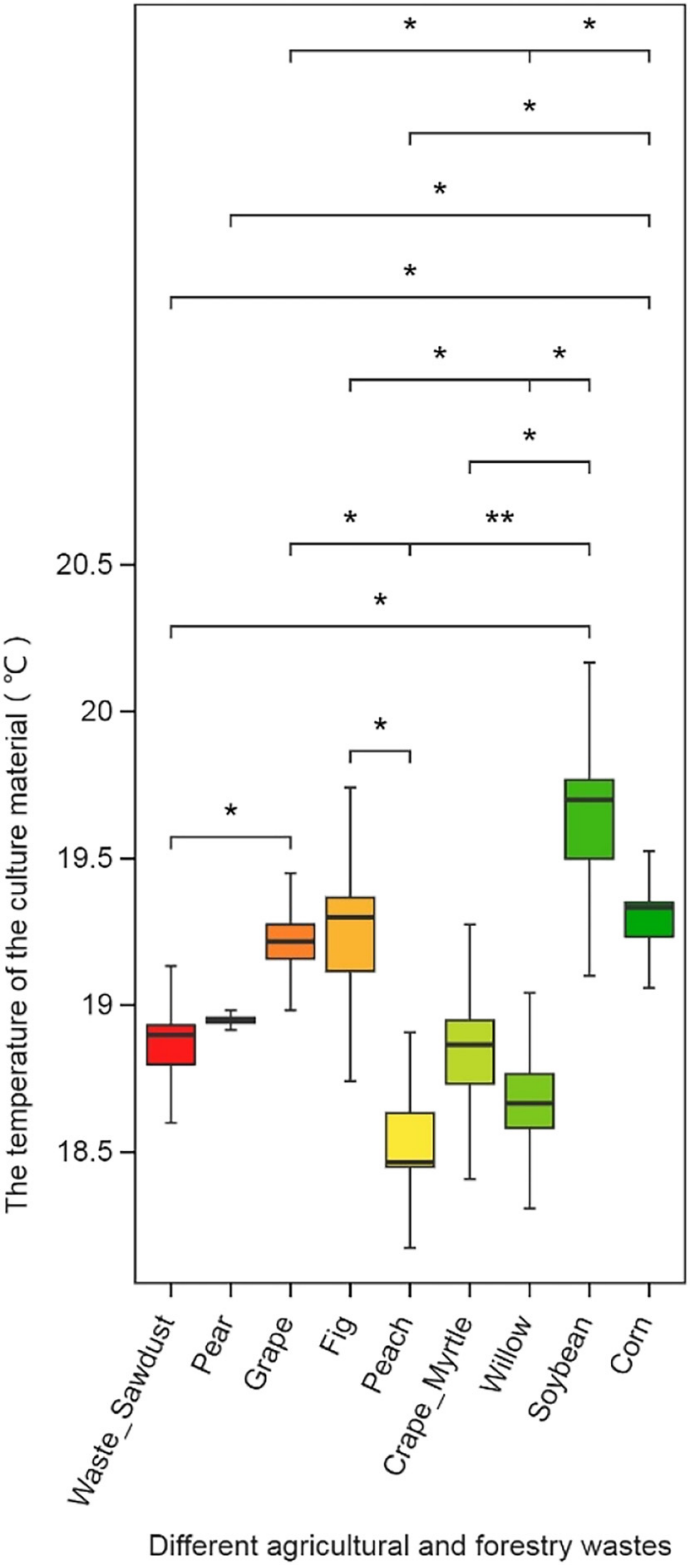
Box diagram of the temperature of *S*. *rugosoannulata* culture material.

As shown in Figure 3, the substrate temperatures were measured 20 days after sowing, with an ambient air temperature of 18.9 °C. Substrates containing waste sawdust, peach branches, crape myrtle branches, and willow branches presented temperatures below the ambient air temperature. In contrast, substrates with pear branches, grape stumps, fig branches, soybean stalks, and corn stalks presented temperatures above the ambient level. Among all the treatments, the soybean and corn stalk substrates maintained relatively high pre-fruiting temperatures, with plot averages of 19.6 °C and 19.3 °C, respectively. Substrates containing peach and willow branches presented significantly lower pre-fruiting temperatures at 18.6 °C and 18.7 °C, respectively.

Mycelia release heat through metabolic activities during growth, and substrate temperature; thus, it serves as an indicator of mycelial activity [25]. Before fruiting, mycelia in substrates composed of grape branches, fig branches, soybean stalks, and corn stalks all showed vigorous activity, with those in fig-branch- and grape-branch-based substrates initiating fruiting earliest.

### 3.7 Effects of agricultural and forestry wastes on the number of fruit bodies in *S*. *rugosoannulata*

Among the nine agricultural and forestry waste substrates used for *S*. *rugosoannulata* cultivation, the fig branches treatment yielded the highest number of grade A fruiting bodies at 23 per/m^2^. In contrast, the crape myrtle branches treatment produced the lowest number at 8.2 per/m^2^. In terms of the proportion of grade A fruiting bodies, the fig branches treatment had the highest percentage at 5.79 %, whereas the crape myrtle branches treatment had the lowest percentage at 2.35 %. For grade B fruiting bodies, the fig branches treatment again showed superior performance, with a value of 374.5 per/m^2^. Compared to the fig branches treatment, the treatments involving waste sawdust, pear branches, grape stumps, crape myrtle branches, willow branches, and soybean straws all produced more than 300 grade B fruiting bodies per/m^2^, which was not significantly different. However, the treatments with peach branches and corn stalks yielded significantly fewer grade B fruiting bodies, with counts below 300 per/m^2^. The cultivation of *S*. *rugosoannulata* on grapevine and fig branches resulted in a higher yield of grade A fruiting bodies. Notably, these two substrates also exhibited the highest water-holding capacity.

The total number of fruiting bodies was largely influenced by the number of grade B fruiting bodies. All treatments except the corn straw treatment produced more than 300 fruiting bodies per m^2^, with the fig branches treatment resulting in the highest yield of 397.5 per/m^2^ (Table 5).

**Table 5:** The number of fruiting bodies of *S*. *rugosoannulata* cultivated from different agricultural and forestry wastes.

Material	Number of grade A fruiting bodies (per/m^2^)	Grade A fruiting body proportion (%)	Number of grade B fruiting bodies (per/m^2^)	Total number of fruiting bodies (per/m^2^)
Waste sawdust	17.8 ± 2.9 abc	5.16	326.8 ± 4.6 abc	344.7 ± 7.6 ab
Pear branches	17.8 ± 0.4 abc	5.40	312.0 ± 41.5 abc	329.8 ± 58.3 ab
Grape stumps	20.5 ± 6.0 ab	5.68	340.5 ± 9.9 abc	361.0 ± 18.4 ab
Fig Branches	23.0 ± 3.5 a	5.79	374.5 ± 16.5 a	397.5 ± 28.3 a
Peach branches	15.0 ± 3.0 bc	4.84	295.0 ± 36.0 bc	310.0 ± 55.2 ab
Crape myrtle branches	8.2 ± 3.5 d	2.35	340.8 ± 42.8 abc	349.0 ± 39.3 ab
Willow branches	12.0 ± 1.0 cd	3.28	354.0 ± 55.3 ab	366.0 ± 55.9 ab
Soybean stalks	15.7 ± 2.7 bc	4.45	337.2 ± 14.3 abc	352.8 ± 14.1 ab
Corn stalks	13.3 ± 1.8 cd	4.47	284.0 ± 5.7 c	297.3 ± 7.4 b

### 3.8 Effects of agricultural and forestry wastes on the average weight of fruiting bodies in *S*. *rugosoannulata*

The average mushroom weight for grade A fruiting body across all the treatments exceeded 30 g/per (Table 6). Treatments supplemented with peach branches and corn stalks yielded grade A fruiting bodies weighing over 40 g/per, whereas those with old grape stumps and willow branches produced grade A fruiting bodies weighing less than 35 g/per. The average mushroom weight was in the following order: corn stalks ≥ peach branches = waste sawdust = pear branches = soybean stalks ≥ fig branches = crape myrtle branches = willow branches = grape stumps (Table 6). For grade B fruiting bodies, the average weight of mushrooms was about 12 g/per mushroom, with treatments containing grape stumps and peach branches producing mushrooms weighing more than 13 g/per mushroom. The treatment with crape myrtle branches yielded the lowest average mushroom weight of 9.46 g/per. Treatments supplemented with grape stumps and peach branches produced significantly heavier mushrooms than those supplemented with waste sawdust with peach branches, resulting in the highest average mushroom weight of 15.07 g/per. In contrast, the average mushroom weight was significantly lower in the crape myrtle branches treatment than in the other treatments, reaching only 10.10 g/per.

**Table 6:** The average weight of *S*. *rugosoannulata* mushrooms cultivated from different agricultural and forestry wastes.

Material	Average weight per grade A fruiting body (g/per)	Average weight per grade B fruiting body (g/per)	Average weight per fruiting body (g/per)
Waste sawdust	38.90 ± 2.30 ab	10.98 ± 0.31 de	12.42 ± 0.14 b
Pear branches	37.37 ± 5.02 ab	12.84 ± 1.20 abc	14.25 ± 2.14 ab
Grape stumps	33.93 ± 2.06 b	13.78 ± 0.04 a	14.90 ± 0.26 a
Fig Branches	36.47 ± 1.40 b	11.38 ± 0.53 cd	12.82 ± 1.00 ab
Peach branches	42.00 ± 5.05 ab	13.67 ± 1.21 ab	15.07 ± 1.71 a
Crape myrtle branches	35.33 ± 5.01 b	9.46 ± 0.92 e	10.10 ± 1.26 c
Willow branches	34.17 ± 2.20 b	11.09 ± 0.64 d	11.85 ± 0.62 bc
Soybean stalks	37.21 ± 4.14 ab	12.11 ± 1.02 bcd	13.23 ± 1.19 ab
Corn stalks	46.39 ± 8.47 a	12.09 ± 0.18 bcd	13.60 ± 0.38 ab

### 3.9 Effects of agricultural and forestry wastes on S’ rugosoannulata yield

**Figure 4:**
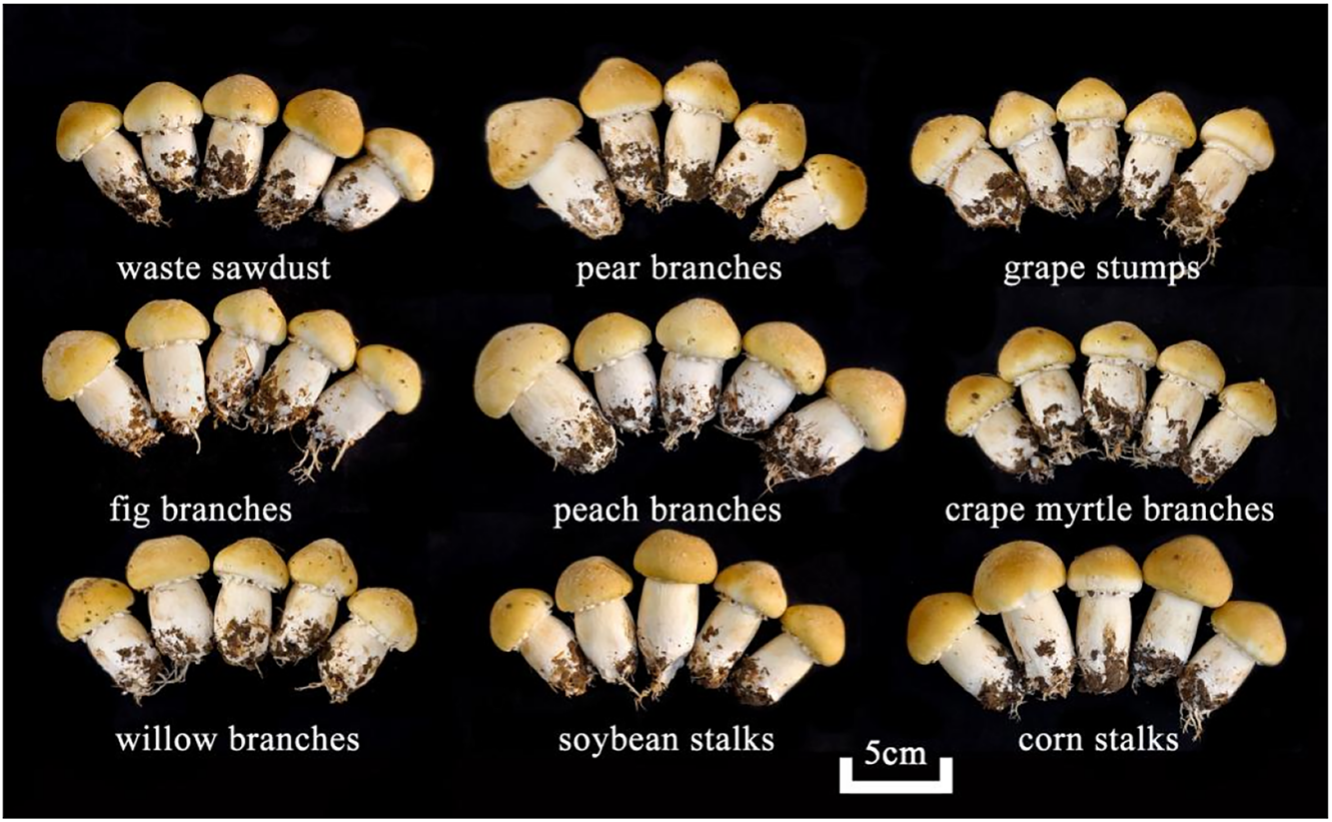
Appearance of the fruiting bodies of the initial tide mushrooms.

The yield of grade A fruiting bodies was the highest in the fig branches treatment at 842.3 g/m^2^, whereas the crape myrtle branches treatment produced the lowest grade A yield at 292.1 g/m^2^ (Table 7). For grade B fruiting bodies, the grape stumps treatment resulted in the highest yield of 4,692.9 g/m^2^, whereas the crape myrtle branches treatment resulted in the lowest yield of 3,203.0 g/m^2^. Compared to the waste sawdust, pear branches, crape myrtle branches, willow branches, and corn stalks treatments resulted in significantly greater total yields exceeding 5 kg/m^2^. The crape myrtle branches treatment resulted in the lowest total yield, at less than 4 kg/m^2^. In terms of the percentage of grade A fruiting bodies yield, the treatments with fig branches and waste sawdust presented the highest percentages, at 16.5 % and 16.2 %, respectively. In contrast, the percentages in the treatments with both crape myrtle and willow branches were less than 10 %. Among the nine agricultural and forestry waste materials tested, fig branches presented the highest conversion rate, at 71.7 %, followed by pear branches, fig branches, peach branches, and soybean stalks. In contrast, waste sawdust, crape myrtle branches, and corn stalks exhibited relatively low biological efficiency, with the crape myrtle branches treatment yielding the lowest efficiency of 46.6 %. Figure 4 illustrates the appearance quality of the first flush mushrooms. The fruiting bodies grown on waste wood, soybean stalks, and corn stalks exhibit more vibrant colors, while those grown on fig and peach branches appear lighter in color.

**Table 7:** Yields of *S*. *rugosoannulata* cultivated from different agricultural and forestry wastes.

Material	Grade A fruiting bodies yield (g/m^2^)	Grade A fruiting bodies yield ratio (%)	Grade B fruiting bodies yield (g/m^2^)	Total yield(kg/m^2^)	BE (%)
Waste sawdust	692.1 ± 109.8 ab	16.2	3,588.8 ± 52.3 cde	4.28 ± 0.06 c	57.1
Pear branches	664.1 ± 102.4 ab	14.3	3,973.3 ± 160.4 bcd	4.64 ± 0.09 bc	61.8
Grape stumps	686.7 ± 245.6 ab	12.8	4,692.9 ± 123.8 a	5.38 ± 0.37 a	71.7
Fig Branches	842.3 ± 212.7 a	16.5	4,268.5 ± 386.9 ab	5.11 ± 0.54 ab	68.1
Peach branches	619.2 ± 102.5 ab	13.4	4,004.6 ± 138.6 bcd	4.62 ± 0.21 bc	61.7
Crape myrtle branches	292.1 ± 151.8 c	8.4	3,203.0 ± 222.8 e	3.50 ± 0.17 d	46.6
Willow branches	409.7 ± 37.2 bc	9.5	3,922.5 ± 633.9 bcd	4.33 ± 0.67 c	57.8
Soybean stalks	582.7 ± 107.6 ab	12.5	4,073.6 ± 180.4 bc	4.66 ± 0.23 bc	62.1
Corn stalks	607.2 ± 30.2 ab	15.0	3,434.3 ± 16.8 de	4.04 ± 0.01 cd	54.0

Most of the treatments did not substantially alter the number of fruiting bodies produced, significant differences in average individual mushroom weight were observed, leading to considerable variations in total yield. The production of grade B mushrooms proved to be a key factor in determining the overall yield. The grape branches treatment excelled due to the greater average weight of its grade B mushrooms, whereas the fig branches treatment benefited from a higher number of fruiting bodies. Consequently, grape branch substrates appear to offer a comparative advantage for cultivating *S*. *rugosoannulata*, particularly in terms of producing larger mushrooms.

### 3.10 Effects of different forestry and agricultural residues on the nutritional composition of fruiting bodies

**Figure 5:**
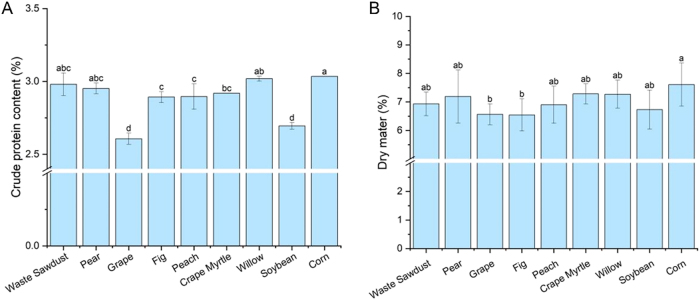
Crude protein (A) and dry matter (B) contents of the fruiting bodies of *S*. *rugosoannulata*.

Fruiting bodies grown on the corn stalk substrate presented the highest crude protein content (3.03 %), followed by those on the willow branch substrate (3.02 %) (Figure 5). A lower crude protein content was detected in fruiting bodies cultivated on grape stumps, soybean stalks, fig branches, and peach branches substrates. The grape stumps and soybean stalks treatments resulted in significantly lower crude protein contents (2.61 % and 2.69 %, respectively). While the dry matter content did not differ significantly across most treatments, fruiting bodies grown on the corn stalk substrate had the highest dry matter content (7.6 %) among the nine waste materials, significantly exceeding those grown on the grape stumps and fig branches substrates. Notably, the fruiting bodies in the grape branch treatment group were poor in quality. This might be attributed to insufficient nutrient accumulation resulting from relatively early fruiting. Additionally, it could be related to certain protein synthesis-inhibiting substances in grape branches, such as heavy metals [26] and specific phenolic compounds [27]. However, this requires further verification.

**Figure 6:**
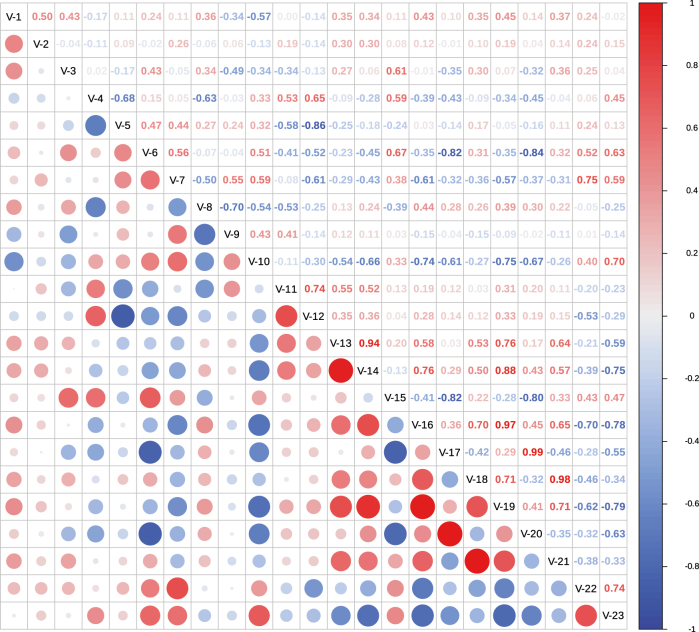
Correlation analysis of all indicators. The variables analyzed included V-1 (water content), V-2 (water holding capacity), V-3 (hemicellulose), V-4 (cellulose), V-5 (lignin), V-6 (total nutrients), V-7 (total carbon), V-8 (total nitrogen), V-9 (substrate C/*N* ratio), V-10 (first harvest), V-11 (harvest day), V-12 (pre-fruiting temperature), V-13 (yield of grade a fruiting bodies), V-14 (number of grade a fruiting bodies), V-15 (average weight per grade a fruiting bodies), V-16 (yield of grade B fruiting bodies), V-17 (number of grade B fruiting bodies), V-18 (average weight per grade B fruiting body), V-19 (total yield), V-20 (total number of fruiting bodies), V-21 (average weight per fruiting body), V-22 (protein content), and V-23 (dry matter content).

### 3.11 Economic benefit accounting

Based on the market average prices, we set the price of grade A mushrooms at 30 yuan/kg and grade B mushrooms at 10 yuan/kg to calculate profit. Labor costs are calculated using the average agricultural labor expense in Jiaxing (120 yuan/person), requiring seven people per hectare for harvesting. The results of the economic analysis are presented in Table 8. When cultivating with fig branches, grade A fruiting bodies of *S*. *rugosoannulata* yielded the highest profit of 138,000 yuan/hm^2^, followed by treatments using waste sawdust, grape stumps, and pear branches, all generating profits exceeding 105,000 yuan/hm^2^ for grade A fruiting bodies. For grade B fruiting bodies, cultivation with grape stumps achieved the highest profit of 233,000 yuan/hm^2^, followed by cultivation with fig branches, soybean stalks, peach branches, pear branches, and willow branches, all yielding profits above 200,000 yuan/hm^2^. The gross profits from crape myrtle branches, willow branches, and corn stalks were lower (below 300,000 yuan/hm^2^). Owing to variations in harvest period, labor costs differed among agricultural and forestry waste substrates. Waste sawdust, soybean stalks, corn stalks, fig branches, and grape stumps incurred labor costs exceeding 70,000 yuan/hm^2^. The formula incorporating peach branches had the lowest labor cost, at 53,000 yuan/hm^2^, whereas the waste sawdust formula had the highest labor cost, at 78,000 yuan/hm^2^. Formulae using fig branches, grape stumps, and peach branches achieved net profits exceeding 200,000 yuan/hm^2^, whereas those using crape myrtle branches and corn stalks yielded lower net profits of 101,000 yuan/hm^2^ and 145,000 yuan/hm^2^, respectively.

**Table 8:** Economic benefits of *S*. *rugosoannulata* cultivated from different agricultural and forestry wastes.

Material	Grade A fruiting bodies profit (thousand yuan/hm^2^)	Grade B fruiting bodies profit (thousand yuan/hm^2^)	Labor cost(thousand yuan/hm^2^)	Gross profit(thousand yuan/hm^2^)	Net profit(thousand yuan/hm^2^)
Waste sawdust	113	196	78	309	166
Pear branches	109	217	65	326	196
Grape stumps	112	256	73	368	230
Fig Branches	138	233	74	371	232
Peach branches	101	219	53	320	202
Crape myrtle branches	48	175	57	223	101
Willow branches	67	214	62	281	154
Soybean stalks	95	222	76	317	176
Corn stalks	99	187	76	286	145

The profits of grade A fruiting bodies and grade B fruiting bodies are calculated using formulas (2) and (3), Labor cost is calculated using formulas (4), Net profit is calculated using formulas (5).

### 3.12 Correlation analysis

A correlation analysis of the above indicators (Figure 6) reveals that the pre-fruiting temperature was positively correlated with the total number of harvest days. Additionally, the pre-fruiting temperature showed a strong negative correlation with the lignin content of the substrate – substrates with higher lignin contents presented lower pre-fruiting temperatures, which correspondingly resulted in shorter total harvest days.

The dry matter content was significantly positively correlated with the protein content, consistent with the findings of Scholtmeijer and Uzun Y et al. [28], 29]. It also showed a significant positive correlation with the first harvest time but a negative correlation with the number of grade A fruiting bodies, yield of grade B fruiting bodies, and total yield. Moreover, the first harvest time was negatively correlated with the total number of fruiting bodies, yield of grade B fruiting bodies, and total yield. Specifically, a shorter first harvest time (i.e., earlier fruiting) led to lower dry matter and protein contents but higher numbers of grade A fruiting bodies, yields of grade B fruiting bodies, total numbers of fruiting bodies, and total yields. Furthermore, the first harvest time could serve as a predictor of the substrate’s yield potential, with materials that fruit earlier being more suitable for cultivation.

The average weight of grade A fruiting bodies was significantly negatively correlated with the total number of fruiting bodies, meaning that fewer fruiting bodies corresponded to greater individual weights of grade A fruiting bodies.

The nutrient content of the substrate was not significantly correlated with total yield and thus cannot be used for yield prediction. However, it was significantly positively correlated with the average weight of grade A fruiting bodies and strongly negatively correlated with the number of grade B fruiting bodies and total fruiting bodies. In other words, substrates with higher nutrient contents produced heavier grade A fruiting bodies but reduced the numbers of grade B fruiting bodies and total fruiting bodies. Therefore, selecting raw materials with higher nutrient content can increase the size of grade A fruiting bodies.

## 4 Discussion

**The influence of the physicochemical properties of the cultivation materials on *S*. *rugosoannulata***. Several studies have demonstrated that the protein content in mushroom fruiting bodies is influenced by substrate chemical composition, carbon-to-nitrogen (C/*N*) ratio, and the specific cultivated species [12]. The C/*N* ratio notably affects mycelial growth, mushroom weight, yield, and fruiting body protein content [30], 31]. However, our correlation analysis revealed no significant associations between substrate nutrient components and unit area yield, suggesting that when different substrate materials are used, nutrient components cannot reliably predict *S*. *rugosoannulata* yield. Instead, changes in substrate physicochemical properties may have a more substantial impact on yield, with particular attention needed to aspects such as heavy metal content, nutrient release efficiency, and particle size.

**Evaluate the applicability of the materials**. Studies have shown that when *S*. *rugosoannulata* can be cultivated with various agricultural and forestry waste materials. These substrates promote earlier initiation of fruiting bodies, generally yield higher quantities of grade B fruiting bodies, and greater overall production per unit area. This pattern can be used to evaluate the suitability of new substrate materials by observing the timing of the first harvest of mushrooms. This relationship was confirmed in studies by Zhou G [31] and Zou Y [32].

**The existing shortcomings and prospects**. Although this experiment classified mushroom fruiting bodies into grades, correlation analysis did not identify factors associated with the yield or quantity of grade A. This limitation may be due to the binary classification system, which resulted in substantial weight differences between grade A and grade B fruiting bodies. This large difference hindered the identification of factors influencing the size of fruiting bodies. Future studies should adopt a more refined grading system.

Although the effects of the decoction on mycelial growth cannot serve as a reliable indicator for determining the suitability of agricultural and forestry waste materials, the finding that certain waste material decoctions enhance mycelial growth presents an intriguing phenomenon. Further studies are needed to identify the specific soluble substances responsible for these growth effects.

Forestry waste materials can be effectively used as growing substrates for various edible mushrooms [33], 34]. In this work, crape myrtle and willow branches performed poorly, suggesting that forestry waste may require fermentation before use or in a reduced proportion if unfermented. Although corn and soybean stalks have relatively low yields, this may be attributed to their herbaceous nature, which renders them prone to decomposition and deficient in sustained nutrient supply; nonetheless, these materials possess distinct advantages.

The branches of fruit trees have a very significant effect on the yield and quality of *S*. *rugosoannulata*. What exactly causes the difference in yield and quality? Further research will be conducted via metabolomics, and the structure of the substrate will be observed to identify the reasons.

## 5 Conclusions

This study assessed the suitability of various raw materials for cultivating *S*. *rugosoannulata*. The timing of the first harvest served as a key indicator of a substrate’s efficacy; earlier first harvests generally suggests a more suitable cultivation medium. Furthermore, incorporating materials with higher lignocellulose contents appeared to positively influence the average weight of individual mushrooms. While all four fruit tree branch substrates yielded favorable production outcomes and hold potential for practical application, grape and fig branches demonstrated particularly promising economic returns, making them highly advantageous choices for cultivation. For shorter cultivation cycles, spent oyster mushroom substrate and corn stalks also presented viable options. Conversely, willow branches, crape myrtle branches, and soybean stalks exhibited less desirable performance, warranting further modification before re-evaluation for cultivation purposes.
